# Diagnosis and Management in Oral and Maxillofacial Surgery

**DOI:** 10.3390/diagnostics16131946

**Published:** 2026-06-23

**Authors:** Dimitris Tatsis, Alexandros Louizakis

**Affiliations:** Department of Oral and Maxillofacial Surgery, Aristotle University of Thessaloniki, 54124 Thessaloniki, Greece; dimitats@auth.gr

Oral and maxillofacial surgery (OMFS) is a rapidly growing specialty, with advances in technology, an improved and deeper understanding of disease biology, and an increasing emphasis on multidisciplinary, patient-centered care. Head and neck region pathologies, including trauma, congenital anomalies, oncological and degenerative disorders, require accurate diagnosis and personalized management to optimize functional and esthetic results. Recent advances have illustrated the potential of artificial intelligence (AI), advanced imaging modalities, three-dimensional (3D) planning and printing, as well as molecular diagnostics and regenerative approaches [1,2].

Despite significant progress, important challenges remain in the field of oral and maxillofacial surgery. The early and reliable detection of pathologies, such as oral squamous cell carcinoma (OSCC) in nontraditional risk groups, such as non-smokers and non-drinkers, the management of rare aggressive lesions in pediatric populations, and long-term functional rehabilitation after complex reconstructions still pose a great challenge. Furthermore, variability in diagnostic approaches, lack of high-level evidence for some interventions, and, finally, the need for better integration of emerging technologies into routine clinical practice continue to impact optimal patient outcomes [3,4].

This Special Issue, “Diagnosis and Management in Oral and Maxillofacial Surgery”, addresses several of these gaps and presents a diverse array of high-quality research. Contributions include comprehensive reviews on rare entities such as pediatric desmoplastic fibroma of the jaws and molecular mechanisms of malignant transformation in salivary gland tumors, clinical studies exploring condylar asymmetry in skeletal anomalies, prognostic modeling in oral cancer incorporating novel nodal parameters, and also practical case reports on surgical challenges such as retrieval of fractured anesthetic needles. The problem is further enhanced by systematic analyses of implant outcome in free bone flap reconstructions and anatomical considerations for procedures such as Le Fort osteotomies. In combination, these works address the importance of combining novel diagnostics (e.g., CBCT-guided approaches and AI potential) with improved surgical techniques and interdisciplinary approaches.

We believe this Special Issue is an important resource that will enhance clinical decision-making and show how modern tools can increase accuracy, reduce complications, and improve long-term outcomes in the OMFS. Hence, various important directions need to be prioritized for future research. Large-scale, multicentre studies in different populations are needed to validate AI-assisted diagnostic tools and patient-specific implants [5]. Further understanding of the etiopathogenesis of OSCC in non-smokers and nondrinkers and progress in the field of regenerative medicine and biomaterials will be essential for prevention and tissue engineering. Molecular biomarkers in salivary gland tumors still represent an unexplored research field that will lead to real changes in the therapeutic protocols for these patients [6]. Longitudinal outcome studies on functional rehabilitation, quality-of-life measures, and cost-effectiveness of new technologies will aid in the translation of innovations into everyday standards of care. Finally, to address disparities in the availability of state-of-the-art OMFS care, it will be critical to foster international collaboration and standard protocols.

We hope that this Special Issue will not only inform current practice but also inspire further innovation and inquiry. The papers presented here are indicative of the dynamism of the field and its commitment to progress in patient-centered diagnostics and management in oral and maxillofacial surgery. We invite readers to interact with these articles and help the continued progress in this important area of healthcare.

## References

[1] Antonelli A., Bennardo F., Giudice A. (2024). Breakthroughs in Oral and Maxillofacial Surgery. J. Clin. Med..

[2] Fiedler M., Off A., Eichberger J., Spoerl S., Schuderer J.G., Taxis J., Bauer R.J., Schreml S., Reichert T.E., Ettl T. (2023). OSCC in Never-Smokers and Never-Drinkers Is Associated with Increased Expression of Tumor-Infiltrating Lymphocytes and Better Survival. Cancers.

[3] Giasimakopoulos P., Mylona D., Diafas A., Stamoulopoulos I., Markou K. (2026). Molecular and Microenvironmental Mechanisms of Malignant Transformation in Benign Salivary Gland Tumors: Implications for Oral Squamous Cell Carcinoma. Diagnostics.

[4] Miragall M.F., Knoedler S., Kauke-Navarro M., Saadoun R., Grabenhorst A., Grill F.D., Ritschl L.M., Fichter A.M., Safi A.F., Knoedler L. (2023). Face the Future—Artificial Intelligence in Oral and Maxillofacial Surgery. J. Clin. Med..

[5] Rana M., Sakkas A., Zimmermann M., Kostyuk M., Schwarz G. (2026). Artificial Intelligence in Oral and Maxillofacial Surgery: Integrating Clinical Innovation and Workflow Optimization. J. Clin. Med..

[6] Yang Z., Du W., Zhang X., Chen D., Fang Q., He Y., Yang Y., Li D., Fan J. (2021). Nonsmoking and Nondrinking Oral Squamous Cell Carcinoma Patients: A Different Entity. Front. Oncol..

